# Novel *KIF6* Polymorphism Increases Susceptibility to Type 2 Diabetes Mellitus and Coronary Heart Disease in Han Chinese Men

**DOI:** 10.1155/2014/871439

**Published:** 2014-12-31

**Authors:** Ge Wu, Gui-Bin Li, Bin Dai, Dong-Qing Zhang

**Affiliations:** Department of Clinical Trials, The Fourth Hospital of Jilin University, Dongfeng Street No. 2643, Changchun, Jilin 130011, China

## Abstract

*Objectives*. The effect of the* KIF6* polymorphism Trp719Arg on the risk of T2DM and T2DM with CHD remains unclear.* Methods*. 946 unrelated subjects of Han Chinese origin were recruited, comprising 346 controls, 312 T2DM, and 288 T2DM + CHD patients. Genotyping was performed by high-resolution melting curve analysis using real-time qPCR. The impact of the variant on T2DM/T2DM + CHD and gene-sex interaction were evaluated by stepwise multiple regression analysis.* Results*. The frequencies of the Trp719 allele in T2DM and T2DM + CHD patients were similar to the control group, whereas significantly increased 719Arg allele frequencies were observed in male T2DM and T2DM + CHD patients compared with the corresponding control group. Further sex partition analysis revealed that only male 719Arg allele carriers had approximately 3-fold and 5-fold higher risk of T2DM and T2DM + CHD, respectively, than noncarriers. There was also a significant association between carriers and higher TG and lower HDL-C levels.* Conclusion*. The KIF6 719Arg allele may increase the risk of T2DM and T2DM + CHD only in Han Chinese men by modulating lipid metabolism, especially with regard to TG and HDL.

## 1. Introduction 

Coronary heart disease (CHD), type 2 diabetes mellitus (T2DM), and T2DM in association with CHD (T2DM + CHD) are multifactorial diseases to whose pathogenesis genetic and environmental factors both contribute. Several environmental factors, such as dyslipidemia, obesity, oxidative stress, smoking, alcoholism, and lack of exercise, have been identified as risk factors for these diseases. In recent years, multiple genetic analysis studies have identified several loci and variants [1–9] that are strongly associated with both T2DM and CHD and have evaluated the predictability of these heritable factors in the development of T2DM and CHD. However, the data to date have explored only a small proportion of the genetic backgrounds of these diseases, leaving behind a large proportion of missing heritability to be unraveled. To discover the common genetic basis of CHD, T2DM, and T2DM + CHD for prompt primary prevention, it is clinically critical to screen out the genetic variants associated with the high risk of developing these diseases.

Kinesin-like protein 6(KIF6), a class of molecular motor from the kinesin 9 superfamily discovered by Vale et al. in 1985 [10], is ubiquitously expressed in the coronary arteries and vascular cells [11] and is involved in intracellular microtubule transportation in association with caveolae [12]. The* KIF6* gene spans a genomic region of about 390,000 base pairs on human chromosome 6p21, containing 23 exons and 22 introns [13]. Since 2007, there have been multiple large prospective and case-control studies on the association of a common* KIF6* gene polymorphism—Trp719Arg single-nucleotide polymorphism (SNP) (rs20455)—with CHD risk [14–23], and a significant relationship between the* KIF6* Trp719Arg polymorphism and CHD has been observed in several ethnic groups [15, 18, 21, 22, 24–26]. Accumulating evidence has suggested that* KIF6* rs20455 may be a new predictive factor for risk of CHD occurrence and a genetic determinant of the plasma lipid level [24, 25, 27–29]. However, it is unknown whether there is an association between* KIF6* Trp719Arg and the occurrence of T2DM/T2DM + CHD.

Both T2DM and CHD are often accompanied by dyslipidemia [30], which has been well defined as an important initial factor for aberrant glucose metabolism with insulin resistance. Hypertriglyceridemia and low high-density lipoprotein cholesterol (HDL-C) are important risk factors for the development of atherosclerosis, the most common cause of death among patients with diabetes. Considering that* KIF6* Trp719Arg is involved in lipid metabolism, we hypothesized that it could have a determinant role in the pathogenesis of both T2DM and CHD. The present case-control study makes an attempt to investigate the significance of the* KIF6* Trp719Arg polymorphism on lipid metabolism in T2DM and T2DM + CHD in a northern Han Chinese population. Our results may add to the evidence on the potential mechanisms of T2DM and T2DM + CHD susceptibility in the Han population beyond the traditional risk factors.

## 2. Experimental Methods

### 2.1. Ethics Statement

During the period of 2011 to 2014, we recruited a total of 946 genetically unrelated Han Chinese subjects from Changchun, Jilin Province of northern China, for the case-control study. Healthy control subjects were recruited by means of advertisement. Subjects of T2DM and T2DM + CHD were all hospitalized patients of the Fourth Hospital of Jilin University, China. Demographic data and baseline clinical information of controls were recorded by internists, and those of the T2DM/T2DM + CHD were obtained by reviewing their medical records. The nature and motif of the study were clearly explained to all participants prior to their signing of full consent forms. The study protocol was approved by the Institutional Review Board of the Fourth Hospital of Jilin University, China.

### 2.2. Study Population

Of the 946 subjects, 312 had T2DM, 288 had T2DM + CHD, and 346 were controls. The body mass index (BMI) was calculated by dividing the body weight in kilograms by body height in meters squared (m^2^). New diabetes was diagnosed based on fasting plasma glucose ≥7.0 mmol/L or oral glucose tolerance test (OGTT) 2 h plasma glucose ≥11.1 mmol/L or both (advocated by the World Health Organization Expert Committee on Diabetes Mellitus in 1999 [31]). Patients with CHD were defined as having >50% stenosis in ≥1 arteries detected by coronary angiography with stable or unstable angina. Hypertension was defined as systolic blood pressure >140 mmHg or diastolic blood pressure >90 mmHg or both or if the patient had a documented diagnosis or was receiving any antihypertensive therapies. Participants were not given lipid-lowering or hypoglycemic medications for at least four weeks prior to the study. The subjects were categorized as smokers (current and former smokers) or nonsmokers and as drinkers (current and former drinkers) or nondrinkers.

### 2.3. Measurement of Blood Glucose, Insulin, and Lipid Levels

Fasting blood lipid, glucose, and insulin concentrations were measured using an automated biochemical analyzer (Hitachi 7170, Tokyo, Japan). Total cholesterol (TC) and triglyceride (TG) were measured by the enzymatic method using reagents supplied by Desay Diagnostic System (Shanghai, China). The HDL-C levels were detected using the direct determination method with reagents supplied by Beijing JiuQiang Biology Technology (Beijing, China). A 75 g OGTT was conducted on all participants. Plasma C-reactive protein (CRP) was measured by an enzyme immunoassay using polyclonal antibodies (Dako, Copenhagen, Denmark). All laboratory parameters were determined in all participants, who had not taken lipid-lowering medication or hypoglycemic agents at least four weeks prior to the study.

### 2.4. Determination of KIF6 Trp719Arg Substitution

Whole blood (5 mL) was drawn into a heparin tube by venipuncture after a 12 h overnight fast. The samples were separated by centrifugation, and aliquots were stored at −86°C until analyses. Genomic DNA was extracted using a DNeasy Blood and Tissue Kit (Qiagen, Valencia, CA, USA). DNA yield and purity were determined by the absorbance ratio measured at 260 nm and 280 nm using a NanoDrop 1000 spectrophotometer (NanoDrop Technologies, Wilmington, DE, USA). Primers were designed according to the United States National Center for Biotechnology Information Primer-BLAST tool. The forward and reverse primer sequences were 5′-CTGTGAAACTCCTTCTG-3′ (17 bp) and 5′-TGGCTTATCAAGAGACATGAGA-3′ (22 bp), respectively. Amplification was performed using 1 *μ*L 10x PCR buffer, 1 *μ*L MgCl_2_ (25 mM), 0.25 *μ*L dNTP (2.5 mmol/L), 0.5 *μ*L EvaGreen saturation dye (20x), 0.25 *μ*L primer (10 *μ*mol/L), 1 *μ*L template DNA (10 ng/*μ*L), 0.1 *μ*L* Taq* enzyme (5 U), and 5.65 *μ*L ultrapure water. Samples were denatured at 95°C for 5 min, followed by 50 cycles at 95°C for 10 s, 60°C for 15 s, and 72°C for 25 s. Final genotyping was performed using high-resolution melt analysis according to the Roche Light Cycler 480 protocol (Roche Applied Science): denaturation of double-stranded PCR product at 95°C for 1 min, annealing at 40°C for 1 min, increasing to 95°C (during the melting period, fluorescence was detected 40 times per second), and then cooling to 40°C for 10 s.

### 2.5. Statistical Analysis

All data were analyzed using SPSS 17.0 (IBM, New York, NY, USA). The clinical and laboratory data were expressed as mean ± SD or percentages. Differences between participant baseline characteristics were assessed by* t*-tests (continuous variables) and chi-square statistics or Fisher's exact test (categorical variables). The outcomes were reported as two-sided *P* values, and *P* < 0.05 was considered statistically significant. Allele frequencies were determined by gene counting. Deviations from Hardy-Weinberg equilibrium and expectations and differences in genotype distributions and allele frequencies between groups were tested by the chi-square test. The associations between T2DM/T2DM + CHD and KIF6 Trp719Arg polymorphism were analyzed by multiple logistic regression analysis with adjustment for individual potential confounders. As a descriptive measure of association between variant and outcome, two-sided *P* < 0.05 was considered statistically significant, and odds ratios (ORs) were calculated along with 95% confidence intervals (CIs). Differences in plasma cholesterol concentrations among genotypes were analyzed with chi-square statistics. The impact of* KIF6* on all outcomes was tested primarily in a dominant genetic model comparing carriers (Arg/Arg, Trp/Arg) to noncarriers (Trp/Trp).

## 3. Results

### 3.1. Baseline Clinical Characteristics

The relevant baseline clinical characteristics, including the traditional risk factors, are summarized in Table 1. Among all variables, age, BMI, smoking status, TG, blood glucose levels, hemoglobin A1c (HA1c), and high-sensitivity CRP (hs-CRP) were significantly higher, and HDL-C was significantly lower in the T2DM/T2DM + CHD groups compared with the controls (all *P* < 0.05).

### 3.2. Frequency Distribution of Genotypes and Alleles

All participants were successfully genotyped for the KIF6 Trp719Arg polymorphism. Univariate analysis was used to determine the genotype and allele frequencies of the KIF6 polymorphism. The genotype distributions were in agreement with Hardy-Weinberg equilibrium (*P* > 0.05). The genotype frequencies were as follows: normal controls: Trp/Trp29.19%, Arg/Trp 47.98%, and Arg/Arg 22.83%; T2DM patients: Trp/Trp 25.64%, Arg/Trp 49.04%, and Arg/Arg 25.32%; T2DM + CHD patients: Trp/Trp 25.69%, Arg/Trp 48.96%, and Arg/Arg 25.35%. There was no significant difference among them (all *P* > 0.05).

Interestingly, further partition analysis of sex showed that, among male subjects, the rs20455 genotypic and allelic frequencies were significantly different between T2DM (*P* < 0.01, Trp/Trp 20.25%, Trp/Arg 51.27%, and Arg/Arg 28.48%, 719Arg allele = 0.5411) and T2DM + CHD patients (*P* < 0.01, Trp/Trp 19.44%, Trp/Arg 52.08%, and Arg/Arg 28.47%, 719Arg allele = 0.5451) compared to the controls (Trp/Trp 30.59%, Trp/Arg 46.47%, and Arg/Arg 22.94%, 719Arg allele = 0.4618). However, these differences were not significant in the female subjects (all *P* > 0.05).

### 3.3. Association of KIF6 Trp719Arg with T2DM and T2DM + CHD Adjusted for Established Risk Factors

Correlations of the SNP with T2DM and T2DM + CHD susceptibility were explored further using the modes of inheritance. In the dominant inheritance mode, binary logistic regression analyses after adjusting for established risk factors demonstrated that 719Arg carriers had a tendency for increased T2DM and T2DM + CHD susceptibility (adjusted OR 1.3321, 95% CI 0.6922–2.3111, *P* = 0.6611; adjusted OR 1.7034, 95% CI 0.8242–2.6969, *P* = 0.4083, resp.) compared with noncarriers (Table 2).

From further data partitioning by sex, dominant model analysis revealed that, among the male subjects, 719Arg carriers had significantly increased risk for both T2DM and T2DM + CHD, with adjusted OR 3.838 (95% CI 0.538–27.385, *P* < 0.01) and 5.213 (95% CI 1.006–27.005, *P* < 0.01), respectively, compared with noncarriers. In contrast, none of these significant differences was identified in the female subjects.

### 3.4. Correlation between Lipid Profiles and KIF6 rs20455 Genotypes

In all participants, including T2DM and T2DM + CHD patients, lipid levels were not significantly different between 719Arg carriers and noncarriers (Table 3).

Further sex-partitioned analysis showed that T2DM males who were 719Arg carriers had significantly elevated TG levels and decreased HDL-C concentrations compared with noncarriers. However, such significant differences were not observed among female participants. In both sexes, TC and LDL-C were not significantly different between 719Arg carriers and noncarriers.

In T2DM + CHD patients, TG levels were significantly higher in 719Arg carriers than in noncarriers (*P* < 0.05). In contrast, TC, LDL-C, and HDL-C levels were not significantly different among the genotypes (all *P* > 0.05).

## 4. Discussion

This case-control study reports the association of the KIF6 Trp719Arg polymorphism with T2DM and T2DM + CHD in a northern Han Chinese population. In our study, despite detecting no association with the KIF6 Trp719Arg polymorphism in either T2DM or T2DM + CHD in the overall study population, further analysis on sex-differentiated association revealed a significantly higher frequency of the 719Arg allele in the men from both the T2DM and T2DM + CHD groups (both *P* < 0.01). Compared to the noncarriers,* KIF6* 719Arg allele carriers had 3-fold and 5-fold higher prevalence of T2DM and T2DM + CHD.

There were significantly higher CRP levels together with larger smoker populations, higher TG, and lower HDL in the T2DM and T2DM + CHD patients. These clinical characteristics well fit the notion that the pathogenesis of T2DM and CHD is associated with inflammation. These determinants serve as nongenetic risk factors for those diseases, while a disease-related polymorphism may serve as a genetic risk factor. It has been reported that* KIF6* 719Arg allele carriers could have increased CHD risk, with male carriers often facing higher CHD risk than females [18, 32, 33]. The Wellcome Trust Case-Control Consortium CHD study demonstrated that the* KIF6* 719Arg allele is associated with increased risk of CHD only in males [34], which appears to be in agreement with our results, where the* KIF6* Trp719Arg polymorphism may only be involved in T2DM and T2DM + CHD development in males as a genetic risk factor for the diseases. Therefore, it may be suggested that the correlation of the 719Arg allele with these diseases may truly be sex-specific.

Dyslipoproteinemia has been identified as an important independent risk factor of both T2DM and CHD. Collectively, genome-wide association studies have identified SNPs in at least 95 loci, the combined effects of which account for approximately 10–12% of the total variance in HDL and TG levels [35]. Previously, we verified that the common* KIF6* polymorphism Trp719Arg is involved in lipid metabolism and CHD, reporting that it was associated with significantly higher TG levels with increased myocardial infarction risk in angiographic CHD patients [24]. The characteristic features of diabetic dyslipidemia are increased concentrations of plasma TG and small dense LDL-C particles and decreased HDL-C [36]. The present results suggest that the 719Arg allele is nominally significantly associated with higher TG and lower HDL-C levels in T2DM and T2DM + CHD male carriers. Hypertriglyceridemia is the most common disorder of lipid metabolism in diabetic patients and is an independent risk factor for T2DM, especially in primary T2DM or patients with poorly controlled blood glucose. Insulin resistance has also been correlated with intracellular TG storage, which is involved in lipotoxicity and beta cell failure leading to diabetes [37]. With the impact on plasma lipid metabolism, the 719Arg allele may accelerate the pathogenesis of T2DM and T2DM + CHD in a sex-specific manner.

T2DM is known as a major independent risk factor for CHD and T2DM patients face a 3-fold higher risk of developing CHD [38]. However, T2DM and CHD pathogenesis share some risk factors. It was recently discovered that most of the active molecules involved in the common risk factors of insulin resistance/hyperglycemia, hyperlipidemia, hypertension, and endothelial dysfunction are colocalized and interact with each other at the cell surface caveolar domains [39]. The* KIF6*-encoded protein kinesin is also involved in the functionality of caveolae [40] and is involved in the intracellular internalization and externalization cycle of caveolae. As the number of caveolae present on the cell surface is vital for cellular nutrient transportation, message signaling, glucose and lipid metabolism, and secretions, the aberrance of any molecule involved can interfere with the cell surface presentation and caveolae functionality, resulting in any combination of those metabolic diseases. Our findings regarding the phenotypes of the* KIF6* polymorphism imply that the encoded kinesin protein loses its original functionality of caveolae interaction, which induces multiple aberrations in glucose and lipid metabolism and in cardiovascular endothelial cell functionality. However, whether such a polymorphism only exerts significant effects on males is still awaiting further investigation.

Multiple genetic analysis studies have suggested that CHD and T2DM may share a common genetic background [2, 41–43]; however, the association between the genetic variability of the* KIF6* Trp719Arg polymorphism and T2DM risks has not been reported thus far. We identified a suggestive association between* KIF6* Trp719Arg polymorphism and T2DM susceptibility. After adjusting for the independent traditional risk factors, 719Arg male carriers had a 3-fold higher risk of developing T2DM compared to noncarriers. Clinical studies have demonstrated that T2DM patients with CHD had shorter life expectancy and profound atherosclerosis progress, with greater vascular stenosis and longer duration of diabetes, and were more prone to developing larger lesions and multivessel disease, with significantly higher occurrence of stent restenosis. Therefore, early prediction is crucial for preventing T2DM complicated by CHD. The present study demonstrated that, after adjusting for the independent traditional risk factors, the 719Arg allele is a major predictor of T2DM + CHD development in male Han Chinese and that male 719Arg carriers face a 5-fold higher risk of developing T2DM + CHD compared with male noncarriers. A similar investigation was conducted by Shi et al. [44] two years before. Based on the results from 300 samples, Shi et al. concluded that there was not significant connection between KIF6 Trp719Arg and T2DM with CHD, which is contrary to the results in this study.

The present study was only a preliminary exploration of the correlation between the* KIF6* Trp719Arg polymorphism and T2DM and CHD, and further functional studies involving more comprehensive and larger samples investigating a heterogeneous association are warranted to elucidate their underlying mechanisms. In contrast to nongenetic clinical markers, genetic markers do not change with time and may be helpful for identifying high-risk subjects, such as for CHD, prior to onset and for enabling the implementation of early preventive programs and for predicting the risk of vascular complications among diabetic male individuals. A description of the full complexity of the genetic background of CHD and T2DM prevalence may become feasible in the near future, which would be of more value for achieving these goals.

## 5. Conclusions

The current study provides the first evidence of a heterogeneous association with sex between T2DM and T2DM + CHD susceptibility and the* KIF6* Trp719Arg variant. With its effect on lipid metabolism, the 719Arg allele may be an independent risk factor for T2DM and CHD in males. Therefore, early intervention for carriers of the 719Arg allele may have extremely vital significance in the occurrence, development, and prevention of T2DM and T2DM + CHD.

## Figures and Tables

**Table 1:** General clinical characteristics of the study participants.

General characteristics	Control (*n* = 1384)	T2DM (*n* = 1248)	T2DM + CHD (*n* = 1152)
Age (years)	**60.23** ± **10.41**	**63.72** ± **9.66** ^∗^	**63.88** ± **10.61** ^∗∗^
Sex (M/F)	680/704	632/616	576/576
BMI (kg/m^2^)	**22.6 ± 2.9**	**25.4 ± 2.8** ^∗^	**26.8 ± 2.4** ^∗∗^
Nondrinker (%)	80.6	78.3	65.4
Current drinker (%)	17.6	20.6	32.5^∗∗^
Former drinker (%)	2.2	2.3	2.3
Nonsmoker (%)	63.1	55.3^∗^	53.0^∗∗^
Current smoker (%)	**32.8**	**40.3** ^∗^	**42.8** ^∗∗^
Former smoker (%)	4.1	4.4	4.2
FPG (mmol/L)	**4.62 ± 1.2**	**9.40 ± 2.4** ^∗^	**10.01 ± 2.4** ^∗∗^
HbA1c (mmol/L)	**4.43 ± 1.1**	**8.12 ± 1.3** ^∗^	**9.46 ± 1.5** ^∗∗^
TC (mmol/L)	4.47 ± 0.92	5.24 ± 1.24	5.72 ± 1.00
TG (mmol/L)	**1.54 ± 0.72**	**2.84 ± 1.25** ^∗^	**3.20 ± 1.43** ^∗∗^
HDL-C (mmol/L)	**1.37 ± 0.21**	**1.24 ± 0.22** ^∗^	**1.11 ± 0.19** ^∗∗^
LDL-C (mmol/L)	2.83 ± 0.75	2.92 ± 0.75	2.85 ± 0.77
hs-CRP	**0.16 ± 0.08**	**1.98 ± 0.42** ^∗^	**5.08 ± 1.03** ^∗∗^

^∗^ *P* < 0.05 comparison between controls and T2DM, ^∗∗^ *P* < 0.05 comparison between controls and T2DM + CHD. BMI, body mass index; FPG, fasting plasma glucose; HbA1c, hemoglobin A1c; TC, total cholesterol; TG, triglyceride; HDL-C, high-density lipoprotein cholesterol; LDL-C, low-density lipoprotein cholesterol; hs-CRP, high-sensitivity C-reactive protein; T2DM, type 2 diabetes mellitus; CHD, coronary heart disease.

**Table 2:** Correlation of T2DM and T2DM + CHD estimated by binary regression analyses.

Groups	*n*	Trp/Trp *n* (%)	Trp/Arg *n* (%)	Arg/Arg *n* (%)	MAF	Dominant model (allelic)		Adjusted log-dominant mode	
						OR (95% CI)	*P* value	OR (95% CI)	*P* value
All									
Control	346	101 (29.19)	166 (47.98)	79 (22.83)	0.4682	1.00		1.00	
T2DM	312	80 (25.64)	153 (49.04)	79 (25.32)	0.4984	1.2154 (0.8613–1.7149)	0.2939	1.332 (0.369–4.811)	0.6611
T2DM + CHD	288	74 (25.69)	141 (48.96)	73 (25.35)	0.4983	1.212 (0.8524–1.7231)	0.3261	1.703 (0.714–7.2969)	0.4083
Males									
Control	170	52 (30.59)	79 (46.47)	39 (22.94)	0.4618	1.00			
T2DM	158	32 (20.25)	81 (51.27)	45 (28.48)	0.5411	**1.7352 (1.0452–2.8807)**	**0.0425**	**3.838 (0.538–27.385)**	**<0.01**
T2DM + CHD	144	28 (19.44)	75 (52.08)	41 (28.47)	0.5451	**1.7755 (1.048–3.008)**	**0.0368**	**5.213 (1.006–27.005)**	**<0.01**
Females									
Control	176	53 (30.11)	86 (48.86)	37 (21.02)	0.4545	1.00		1.00	
T2DM	154	48 (31.17)	72 (46.75)	34 (22.08)	0.4545	0.9516 (0.5953–1.521)	0.9048	0.934 (0.304–2.875)	0.9053
T2DM + CHD	144	46 (31.94)	66 (45.83)	32 (22.22)	0.4514	0.918 (0.5704–1.4774)	0.8080	0.985 (0.564–1.720)	0.9582

OR = odds ratio; 95% CI = 95% confidence interval; MAF = minor allele frequency; allele frequencies were estimated by direct counting. ^∗^Adjusted for age, BMI, smoking status, alcohol status, hypertension, TC, LDL-C, and hs-CRP. T2DM, type 2 diabetes mellitus; CHD, coronary heart disease.

**Table 3:** Associations of lipid levels with different genotypes of both genders.

Groups	TC			TG			HDL-C			LDL-C		
	Control	T2DM	T + CHD	Control	T2DM	T + CHD	Control	T2DM	T + CHD	Control	T2DM	T + CHD
Trp/Trp	4.45 ± 1.011	5.21 ± 1.31	5.69 ± 0.92	1.55 ± 0.69	2.71 ± 1.3	3.12 ± 1.36	1.41 ± 0.23	1.26 ± 0.17	1.16 ± 0.15	2.8 ± 0.76	2.85 ± 0.69	2.78 ± 0.74
Trp/Arg + Arg/Arg	4.49 ± 0.829	5.27 ± 1.17	5.75 ± 1.08	1.53 ± 0.75	2.97 ± 1.2	3.28 ± 1.5	1.35 ± 0.19	1.21 ± 0.27	1.12 ± 0.23	2.85 ± 0.74	2.99 ± 0.81	2.92 ± 0.8
Males												
Trp/Trp	4.49 ± 1.02	5.26 ± 1.34	5.69 ± 0.99	1.55 ± 0.81	2.54 ± 1.12	2.98 ± 1.42	1.41 ± 0.19	1.25 ± 0.22	1.15 ± 0.16	2.86 ± 0.77	2.8 ± 0.72	2.82 ± 0.72
Trp/Arg + Arg/Arg	4.53 ± 0.91	5.34 ± 1.28	5.8 ± 1.11	1.62 ± 0.72	3.22 ± 1.25^a^	3.46 ± 1.39^b^	1.34 ± 0.22	1.15 ± 0.18^a^	1.09 ± 0.23	2.9 ± 0.75	3.04 ± 0.74	3.01 ± 0.81
Females												
Trp/Trp	4.41 ± 1.00	5.16 ± 1.28	5.69 ± 0.85	1.55 ± 0.57	2.88 ± 1.48	3.26 ± 1.3	1.41 ± 0.27	1.27 ± 0.12	1.17 ± 0.14	2.76 ± 0.75	2.9 ± 0.66	2.74 ± 0.76
Trp/Arg + Arg/Arg	4.45 ± 0.74	5.2 ± 1.06	5.7 ± 1.05	1.44 ± 0.78	2.72 ± 1.15	3.1 ± 1.61	1.36 ± 0.16	1.27 ± 0.36	1.15 ± 0.23	2.8 ± 0.73	2.94 ± 0.88	2.83 ± 0.79

TC, total cholesterol; TG, triglycerides; HDL-C, high-density lipoprotein cholesterol; LDL-C, low-density lipoprotein cholesterol; T and T2DM, type 2 diabetes mellitus; CHD, coronary heart disease.

^a^ *P* < 0.05, ^b^ *P* < 0.01 versus the control.
